# Clinical and Molecular Aspects of Vitiligo Treatments

**DOI:** 10.3390/ijms19051509

**Published:** 2018-05-18

**Authors:** Anuradha Bishnoi, Davinder Parsad

**Affiliations:** Department of Dermatology, Venereology and Leprology, Post Graduate Institute of Medical Education and Research, Sector 12, Chandigarh 160012, India; dranha14@gmail.com

**Keywords:** vitiligo, stabilizing treatments, repigmenting treatments

## Abstract

Vitiligo is an asymptomatic but cosmetically disfiguring disorder that results in the formation of depigmented patches on skin and/or mucosae. Vitiligo can be segmental or non-segmental depending upon the morphology of the clinical involvement. It can also be classified as progressing or stable based on the activity of the disease. Further, the extent of involvement can be limited (localized disease) or extensive (generalized disease). The treatment of vitiligo therefore depends on the clinical classification/characteristics of the disease and usually comprises of 2 strategies. The first involves arresting the progression of active disease (to provide stability) in order to limit the area involved by depigmentation. The second strategy aims at repigmentation of the depigmented area. It is also important to maintain the disease in a stable phase and to prevent relapse. Accordingly, a holistic treatment approach for vitiligo should be individualistic and should take care of all these considerations. In this review, we shall discuss the vitiligo treatments and their important clinical and molecular aspects.

## 1. Introduction

Vitiligo is an asymptomatic but cosmetically disfiguring disorder that results in the formation of depigmented patches on skin and/or mucosae [1,2]. Vitiligo can be segmental or non-segmental depending upon the morphology of the clinical involvement [3]. It can be classified as progressing or stable based on the activity of the disease. Further, the extent of involvement can be limited (localized disease) or extensive (generalized disease). The treatment of vitiligo therefore depends on the clinical classification/characteristics of the disease and usually comprises of 2 strategies. The first involves arresting the progression of active disease (to provide stability) in order to limit the area involved by depigmentation. The second strategy aims at repigmentation of the depigmented area. It is also important to maintain the disease in a stable phase and to prevent relapse [4]. A holistic treatment approach for vitiligo should therefore be individualistic and should take care of all these considerations. In this review, we shall discuss the vitiligo treatments and their important clinical and molecular aspects.

A multitude of plausible theories have been put forward to explain the pathogenesis of vitiligo and mechanisms that finally lead to the loss of functional melanocytes from the epidermis. The important ones include a genetic predisposition, autoimmune destruction of melanocytes, altered redox status and free radical mediated melanocyte damage, heightened sympathetic response and catecholamines/neurotransmitter mediated melanocyte damage, and impaired melanocyte adhesion or melanocytorrhagy. The combination of all these effectively explains the vitiligo pathogenesis (the combination theory) [5]. The treatment of vitiligo comprises medical, phototherapeutic and surgical modalities. In some situations, a combination of these methods works the best [6]. The aim is to prevent the ongoing destruction of melanocytes and to provide mediators that can stimulate the growth and proliferation of existing melanocytes, resulting in successful repigmentation.

## 2. Stabilization Treatments

Firstly, we will discuss the stabilization therapies. Active vitiligo is characterized by trichrome appearance, confetti like lesions, rare inflammatory margins and Koebner’s phenomenon [7]. The presence of Koebner’s phenomenon also correlates with greater body surface area involvement and poor response to treatment. Stabilization therapies help in halting the progression of ongoing active disease and are usually accompanied by some degrees of simultaneous repigmentation, though it varies amongst studies with some reporting significant, and some reporting minimal repigmentation [8,9,10].

The autoimmune theory is one of the most plausible theories that have been proposed to understand the pathogenesis of vitiligo [5]. Moreover, many other theories finally converge on the autoimmune destruction of melanocytes. Vitiligo has been found to be associated with other prototype autoimmune diseases including alopecia areata, Hashimoto thyroiditis, pernicious anemia and type 1 diabetes mellitus [5,11,12]. Genome-wide association studies have deciphered about 50 susceptibility loci for vitiligo, and the majority of these (about 90%) confer to innate and adaptive immunity and the rest (about 10%) confer to melanocyte antigens and stress-response pathways [13]. Overall, vitiligo has been thought of as a polygenic trait [14,15,16].

Though neural theory gained a significant credence previously, especially to explain the pathogenesis of segmental vitiligo, it has been seen recently that the distribution in segmental vitiligo is rarely confined to the neural pattern. The distribution is rather mosaic and conforms to the affected melanocytes in that localized segment (rather than the neural elements) and can present as a linear, phylloid, block like and checker board pattern. In view of significant interface dermatitis that was demonstrated in a patient having segmental vitiligo, the focus has now shifted to autoimmunity in segmental vitiligo as well [12].

Autoimmunity can be humoral or cellular. Though the research pertaining to the pathogenesis of this intriguing disease continues, the evidence garnered over the time suggests a more crucial involvement of cellular immunity in vitiligo, whereas, humoral immunity probably plays a secondary part, wherein antibodies are produced against the fractions/antigens of melanocytes that are produced/released during the process of active melanocyte destruction by cellular immunity like tyrosinase, tyrosinase like protein 1 and 2, glycoprotein 100 (gp100) and melanoma antigen recognized by T cells (*MART-1)*, all of which are specific melanocyte antigens [17]. Three key players in autoimmune damage characteristic of vitiligo are melanocytes, immune effector cells and environmental triggers [18].

CD8^+^ cytotoxic T cells are considered the most important mediators of melanocyte injury taking place in the active disease [19]. Their capability of causing melanocyte destruction has been demonstrated in many in vitro studies. The ratio of CD4^+^ /CD8^+^ cells is found to be increased in the peripheral blood of vitilig*o* patients. Further, it has also been observed that owing to significant skin homing, the levels of CD8^+^45RO^+^/CD8^+^CLA^+^ cells decline in the peripheral blood [20]. It has been consistently seen that the patients responding poorly to the treatment have enhanced CD8^+^ and CD45RO^+^ cells in their perilesional skin [21]. The degree of perilesional infiltrate directly correlates with the activity and severity of the disease [7]. It has been observed that the presence of CD8^+^ cells in the perilesional skin correlated strongly with a poor response to treatment, including surgical grafting. Levels of interleukin 1 (IL-1) and IL-12 have been found to be increased in the blister fluid obtained from the lesional skin in patients having active and treatment refractory disease. Vitiligo has been seen to develop in recipients after bone marrow transplantation from donors having vitiligo [18]. These findings provide credence to the involvement of CD8^+^ cytotoxic T-cells in the pathogenesis of vitiligo.

These melanocyte-specific cytotoxic T-cells are thought to be both sufficient and necessary to cause melanocyte destruction [22]. The usual mechanism of cytotoxic T-cell mediated cellular damage is via the involvement of Fas-Fas ligand, perforins and granzyme. In vitiligo, interferon gamma (IFN-γ) has been demonstrated to be an important mediator for the actions of cytotoxic T-lymphocytes (similar to lichen planus and systemic lupus erythematosus). Overall, the T-helper 1 (Th1) pathway is considered quite significant in vitiligo, and these T-cells are thought to produce elaborate amounts of IFN-γ that further acts on the IFN-γ receptors.

Important in this IFN-γ signaling cascade are the intracellular janus kinases (JAK) and signal transducers and activators of transcription (STATs). JAKs are intracellular, non-receptor associated tyrosine kinases (JAK 1, 2, 3 and *TYK* 2). Once a cytokine binds to its receptor, JAKs are activated and with the help of adenosine triphosphate (ATP), undergo autophosphorylation. Further, they cause phosphorylation of receptors and induce conformational changes in the form of docks for STATs to bind. After STATs bind to the docks inside the receptors, JAKs carry out phosphorylation of STATs, which in turn activates these STATs.

The activated STATs then translocate inside the nucleus and act as transcription-activators and result in the transcription of multiple mediators that carry out the functions of the bound cytokine. These JAK-STATs are located inside many cells and can carry out the functions of multiple cytokines. Therefore, their inhibitors, despite specifically acting against JAK-STATs, can affect the mediation of a broad array of cytokine network inside many cells. Therefore, as opposed to traditional biologics, which act against a particular cytokine, the target of action of these JAK-STAT inhibitors becomes quite broad.

JAKs located inside keratinocytes are activated once IFN- γ binds to a heterodimeric receptor on a keratinocyte [23]. Their activation is followed by translocation of STAT1 to the nuclear domains, where it activates the transcription of early to intermediate IFN-γresponsive genes that include *CXCL9* and 10. *CXCL9* and 10 are potent chemokines that act on receptor CXCR3 present on T-cells and result in further recruitment and homing of cytotoxic T-cells in the vitiligo skin [19,24]. These T-cells then produce more IFN-γ and the vicious cycle thus continues. Moreover, the perilesional infiltrate also contains the plasmacytoid dendritic cells, which secrete IFN-alpha, and IFN-alpha causes further chemotaxis of cytotoxic T-lymphocytes to the lesional skin.

The expression of IFN-γ and the downstream genes has been found to be enhanced in the vitiligo skin. It has been observed that IFN-γ directly inhibits melanogenesis (functional or qualitative inhibition) and results in the apoptosis of the melanocytes (quantitative inhibition). It induces senescence of melanocytes and promotes the release of heat shock protein −70 which marks the melanocytes for damage by innate immune response [25]. The reasons for selective targeting of melanocytes by IFN-γ remain unclear and may be due to selective up-regulation of the IFN-γ receptor on melanocytes. To conclude, IFN-gamma directly causes melanocyte apoptosis and also enhances the active influx of activated T-cells in the skin via chemokines released from the keratinocytes, causing a vicious cycle that culminates in depigmented patches on skin. The inhibition of this IFN-γ signaling can have an important impact on the management of vitiligo.

JAK-STAT inhibitors have shown promising results in the treatment of vitiligo, including successful repigmentation outcomes. These molecules have been tried in both topical and systemic formulations. Pan JAK inhibitor, tofacitinib and JAK-1,2 inhibitor, ruxolitinib, have been found successful in causing repigmentation in vitiligo [26]. Similarly, statins (which are also HMG co-reductase inhibitors) were demonstrated to inhibit STAT1 signaling in ex-vivo studies, and simvastatin was found to result in significant repigmentation in a case report. Further, though phase 2 clinical trials are going on in ulcerative colitis, the inhibitors of CXCL-10 and CXCR3 could be tried further in vitiligo.

Tofacitinib citrate acts on the main pathogenetic pathway in vitiligo. It has been observed recently that though the administration of tofacitinib effectively abolished the inflammatory response in the lesions (as seen by the measurement of the lesional cytokine levels), repigmentation was observed significantly more on the sites exposed to sun or which received NB-UVB (typically requiring lower-levels of light as compared to routine phototherapy), suggesting that light is the primary stimulator for melanocytes and is essential to induce repigmentation even if the activity of the disease is controlled by immunomodulators [27].

Classical immunosuppressants and immunomodulators probably act on these T-lymphocytes and inhibit their activation and proliferation. Methotrexate, azathioprine and systemic corticosteroids (prednisolone, dexamethasone, betamethasone) have been successfully employed to halt the progression of active vitiligo, though repigmentation is variably reported in different studies [9,28,29,30]. Induction of T-cell inhibition effectively halts the progression of the disease. However, approximately 40% of the patients relapse once these treatments are withdrawn, which points towards a role of memory T-cells residing in the skin that are responsible for the relapse of the disease. The effective inhibition of these memory T-cells can be achieved by employing topical calcineurin inhibitors including tacrolimus during the periods of remission. Topical corticosteroids are the mainstay of treatment for localized disease and have been shown to have a reservoir effect that lasts for around 5 days. The risk of development of glaucoma or cataracts in patients using periorbital topical corticosteroids was not found to be raised in case-control studies [31]. Topical vitamin D analogues have been used with variable outcomes in vitiligo, and the plausible mechanism of action includes immunomodulatory and melanocyte stimulating activities. Though attempts have been made to identify the involvement of other cytokines including TNF-α, IL-17 and IL-6, their role is uncertain and these have not been found to be important as far as pathogenesis of vitiligo is concerned. Instead, vitiligo has been seen to develop de-novo or to exacerbate after institution of anti TNF-α agents.

Why melanocytes are exclusively targeted by the ongoing immune-system activation remains an everlasting question. Though some patients exhibit a rare inflammatory vitiligo in the form of erythema, scaling and pruritus in the borders of the lesions (which is characterized by interface dermatitis histopathologically and may show vacuolated keratinocytes and melanocytes on electron microscopy) [32], most of the times, the disease presents with clinically quiescent depigmented patches that are characterized by loss of melanin and melanocytes [33]. It is thought that some intrinsic defects in the melanocytes of patients with vitiligo render them more susceptible to damage from ongoing cellular metabolism than melanocytes from their healthy counterparts.

Cells constantly face oxidative stress in their immediate environment [34]. This stress can be the result of cellular metabolism and may also result from the exposure to certain environmental toxins. Many theories have been proposed to explain this inherent susceptibility of vitiligo melanocytes to oxidative stress [28]. In vitro studies have revealed that oxidative stress and lipoperoxidative damage leads to reduced expression of E-cadherins, even in normal melanocytes [35]. E-cadherin is a calcium-dependent adhesion molecule that binds the melanocytes to surrounding keratinocytes [36]. Integrins bind melanocytes to the basement membrane. The expression of E-cadherin in melanocytes is normally lower when compared to that in keratinocytes [37]. Exposure to stress reduces the expression of E-cadherin further. The reduced expression of E-cadherin disrupts melanocyte-keratinocyte contact, uproots the melanocytes from their basal location and these start moving upwards in the epidermis [38]. Even the melanocytes in nonlesional skin have been found to be displaced upwards in the epidermis. This upward movement results in the termination of survival signals that melanocytes receive from basement membrane and causes the onset of apoptosis. The markers of apoptosis including phosphatidylserine have been found to be present on melanocytes moving upward in the epidermis. Further mechanical stress in the form of routine frictional forces results in the removal of these melanocytes from the epidermis (melanocytorrhagy) [39]. In fact, studies have revealed reduced and disorderly distributed E-cadherin in the melanocytes obtained from clinically normal looking skin of vitiligo patients [35]. This suggests that all of the skin is actually involved in non-segmental vitiligo, and routine mechanical stresses and friction causes the shedding of these E-cadherin-deficient melanocytes [38]. The credence to this hypothesis is provided by the localization of vitiligo on sites exposed to maximum friction, like knees, elbows, ankles and acral areas. This might also explain the treatment resistance of these sites, since the deficient E-cadherin cannot be replenished, and even if the melanocytes are transplanted from seemingly normal skin, these shall harbor the E-cadherin deficiency and shall fall off on exposure to mechanical stresses. This also provides importance to the maintenance of the redox status inside cells and the significance of antioxidants, since they might help [35] in reducing the damage to E-cadherin that is sustained by the lipoperoxidative stress.

It has also been seen that E-cadherin-deficient mice did not develop vitiligo until their tails were repeatedly brushed, signifying the role of mechanical friction and trauma in the causation and progression of vitiligo [40]. A recent study has revealed a significant reduction in the amount of laminin and integrin in vitiligious skin as compared to nonlesional skin from the same vitiligo patients. The study also revealed reduced intercellular adhesion molecule-1 and vascular cell adhesion molecule-1, though these were not significant. Notably, no difference was found between vitiliginous and nonlesional skin regarding the levels of beta catenin, E-cadherins and collagen 1V [41]. This might be because deficiency of E-cadherin is found to be uniformly distributed between lesional and nonlesional skin [38]. The polymorphism of the gene CDH1 that encodes E-cadherin was recently studied in vitiligo. Not only was the polymorphism found to be significant for vitiligo, it was also found to be associated with autoimmune comorbidities [37]. Also, in a study where E-cadherin expression was compared between vitiligo skin and controls, the expression of E-cadherin inside keratinocytes was found to be normal in vitiligo keratinocytes, but the expression inside melanocytes was significantly lower. Moreover, an inverse correlation was found between E-cadherin expression and infiltration of T-lymphocytes. What remains to be seen is that if loss of E-cadherin (secondary to oxidative stress) is the primary event in vitiligo, how does it stimulate the immune response? Is it the loss of E-cadherin or the upward displacement and subsequent apoptosis of melanocytes that results in the expression of melanocytic antigens on their surface and subsequent immune response? [37]. Also, though strategies are available to ameliorate oxidative stress, it remains to be seen if replenishment of E-cadherin could be employed in localized vitiligo.

The melanocytes in vitiligo are thought to be more prone to develop damage from ‘unfolded protein response’ (stress signals generated as a result of formation of misfolded/unfolded proteins) and free radical generation. Melanogenesis is a major metabolic pathway and requires the synthesis of a large amount of proteins to form tyrosinase and other related enzymes [42]. Some proteins formed during this process are not folded properly in a tertiary or quaternary configuration and are subsequently eliminated. A genetic predisposition might exist in vitiligo melanocytes that leads to the formation/accumulation of altered or misfolded tyrosinase and related enzymes. The process of melanogenesis involves significant hydroxylation and oxidation and consequently leads to the formation of free radicals [43]. The lack of adequate free radical scavenging machinery renders melanocytes susceptible to membrane and nuclear damage by these reactive molecular species and generates stress in melanocytes [28].

Trauma, stress, infection, malignancy, neural dysfunction, vaccination, pregnancy, drugs, xenobiotics, pollution, and calcium imbalance either by themselves or in the presence of abnormal mitochondria with an abnormal distribution of membrane lipids, cardiolipin and cholesterol, lead to the generation of reactive oxygen species (ROS) in vitiligo. Though previous studies have shown an increased concentration of epidermal hydrogen peroxide, the recent findings seem contrary [44]. Reduced expression of catalase, and an enhanced expression of superoxide dismutase and consequent increased levels of malondialdehyde and DNA-peroxidation products have been described in many previous studies and established in a recent meta-analysis [45]. Another recent meta-analysis also shows that the levels of glutathione peroxidase are low in vitiligo [46]. Therefore, melanocytes in vitiligo have enhanced sensitivity to oxidative stress because of reduced expression of catalase and glutathione peroxidase, 2 enzymes that are known to metabolize the ROS including hydrogen peroxide, hydroxyl ion, superoxide and singlet oxygen [47].

A specific polymorphism, 389 C/T in the catalase gene, has been previously described in vitiligo, but was found to be not significantly associated in a recent meta-analysis [48]. The critical antioxidant system, nuclear factor E2 related factor 2-antioxidant response element-heme oxygenase 1 (NRF2/ARE/HO1) has been found to be deficient in disease-free epidermis of vitiligo [49]. This antioxidant system is the chief salvage pathway that protects melanocytes from the oxidative damage induced by hydrogen peroxide. The oxidative damage thus sustained results in apoptosis and vacuolization of melanocytes and keratinocytes. There is a reduction in the melanocyte growth factor and stem cell factor, normally secreted by keratinocytes. Also, inherent ultrastructural abnormalities including abnormal mitochondria, melanosomal compartmentalization and dilated endoplasmic reticulum also predispose melanocytes to more damage by these ROS. Apart from being capable of causing direct cell death and apoptosis, the subcytotoxic stress resulting from oxidation excess can also cause activation of the mitogen-activated protein kinase pathway, Akt pathway and induction of p-16 and p-53-mediated damage in melanocytes. There is formation of DNA-damage products like 8-hydroxy 2-deoxyguanosine during oxidative damage, and it has been seen previously that polymorphism in apyrimidinic endonuclease-1, the enzyme that repairs this DNA damage, can predispose a person to the development of vitiligo [48]. However, the chief autoantigens are derived from melanosomal proteins rather than DNA damage. Tyrosinase and Melan-A containing exosomes are released from melanocytes after exposure to monobenzone and act as autoantigens. An interesting study showed the presence of viable melanocytes in the depigmented skin of vitiligo, which had been stable for as long as 25 years. Moreover, the structural abnormalities including vacuolization and granulation in keratinocytes and melanocytes and dilatation of endoplasmic reticulum completely disappeared after application of phototherapy activated pseudocatalase [50].

This abnormal stress response in melanocytes, apart from causing the reduction of E-cadherin, as discussed above, also marks them for damage by innate immune mechanisms and perpetuates further autoimmunity [51]. It has been observed that calreticulin, a calcium containing intracellular endoplasmic reticulum molecule, localizes to the cell surface and helps in putative antigen presentation to dendritic cells and helps in breaking immune tolerance [48]. Natural-killer cells and inflammatory dendritic cells represent innate immunity. After exposure to melanocytes marked by damage signals, these cells migrate to local lymph nodes and help in the activation of adaptive immunity by presentation of melanocyte specific antigens to naïve T cells and simultaneous production of inflammatory cytokines. This serves as a base for further destruction of melanocytes by cell-specific immunity.

Antioxidants administered either topically or systemically have shown promise though results are variable [52,53]. Polypodium leucotomos, ginkgo biloba [54], pseudocatalase, khellin, vitamin C, vitamin E and polyunsaturated fatty acids like alpha-lipoic acid have been employed with variable outcomes in previously published studies. Minocycline has been found to be beneficial in vitiligo in a series of studies. The effect stems from its successful anti-oxidant role [55]. It has been observed in some studies that hydrogen peroxide accumulates in the epidermis of vitiligo lesions. Many in-vitro studies demonstrate that hydrogen peroxide induces degeneration of melanocytes by activating c-jun N-terminal kinase (*JNK*), p38-mitogen activated protein kinase (*MAPK*) and executioner caspase 3. Minocycline is an antibiotic that decreases the degeneration of melanocytes by directly causing inhibition of these pathways [56].

The stress in melanocytes can also be precipitated in genetically susceptible individuals by the exposure of chemicals like para-tertiary butyl phenol, para-tertiary butyl catechol, hydroquinone and rhododendrone, which bear potential chemical similarity to tyrosine and phenylalanine (the substrates of melanogenesis). These are stipulated to cause vitiligo by competitive inhibition as well as enzyme modifying properties via adduct formation that render tyrosinase immunogenic. The depigmentation occurring after exposure to these chemicals is referred to as chemical leukoderma. Though it initially starts at the site of contact with these chemicals, the later spread and characteristics are identical to idiopathic vitiligo [57]. Additionally, hair dyes and imiquimod have recently been implicated in the causation of chemical leukoderma. Whereas hair dyes probably act by competitive inhibition of tyrosinase by virtue of a wide array of phenolic compounds contained in them, imiquimod induces a local IFN-alpha response that most probably acts on surrounding cells in a way similar to IFN-γ via JAK-STAT pathway. Monobenzyl ether of hydroquinone has been utilized in the management of universal vitiligo with the principle that it will cause depigmentation of body areas with remaining normal pigmentation, thus providing a uniform depigmentation and cosmetic outcome for individuals suffering from universal vitiligo (>80% body surface area involvement).

The stress response in melanocytes also leads to the production and translocation of heat-shock proteins (HSP, notably HSP 70i) to the cell surface where these help in presenting antigens to dendritic cells. These HSPs cause the release of *TRAIL* [18] (TNF alpha related apoptosis inducing ligand), which causes sensitization of the innate immune response and stimulation of dendritic cells, which further leads to the cell-mediated immune response as discussed above. A mutant HSP 70i has been therapeutically employed to reduce dendritic cell stimulation and subsequent T-cell response. *NLRP-*3 (NLR family pyrin domain containing 3) was demonstrated to be an important receptor for inflammasome-mediated melanocyte damage on exposure to chemical stress. This receptor is linked to downstream interleukin 1-β production whose levels have been found to be raised in perilesional vitiligo skin. The modulation of this NLRP-3 and interleukin 1-beta pathway remains to be tried in vitiligo [58].

The consensus regarding the role of T-regulatory cells (Tregs) in vitiligo is still evolving. It has been seen that a repigmentation response associated with narrow-band ultra-violet B (NB-UVB) irradiation correlates with an increase in the level of cytokines released by Tregs, like transforming growth factor beta (TGF-β) [57], though other studies did not find a correlation between the activity of vitiligo and the presence of Tregs. Another interesting finding is the presence of CD8+ resident memory T-cells in both active and stable vitiligo, which might lead to relapses and flares in the disease activity of vitiligo and can be therapeutically targeted [59]. Levels of IL-17 and IL-1β were found to be raised in the sera and epidermis of active vitiligo patients when compared to stable patients, and this increase was associated with decreased levels of microphthalmia associated transcription factor, a melanocyte augmenting factor [58]. The expression of liver-x receptor alpha was also found to be increased in the perilesional skin of vitiligo, and it correlated with a reduction in matrix metalloproteinase activity, which is required for repigmentation. Inhibition of this molecular target might be of significance in the induction of repigmentation in vitiligo [60].

Finally, the role of dermal fibroblasts in augmenting depigmentation has recently been studied. It has been observed that genetically altered fibroblasts in vitiligo patients contribute to melanocyte loss by producing tenascin and dickkopf-1, two molecules that impair the adhesion of melanocytes to surrounding keratinocytes and facilitate the process of melanocytorrhagy [61]. Dickkopf 1 has also been found to induce melanocyte senescence [62,63]. It has been postulated as one of the reasons why acral areas will not repigment easily. Apart from a reduced number of basal melanocytes and lack of hair follicles, the dermal fibroblasts in these sites have been shown to release more tenascin and dickkopf-1 in comparison to other sites. Additionally, similar to the senescence of melanocytes observed in the normal skin of vitiligo patients, a specialized type of senescent fibroblast, myofibroblast, has been found in the dermal compartment of vitiligo patients that leads to abnormal dermal-epidermal talk in vitiligo and contributes to progressive vitiligo. Though few studies have shown that the dermal mesenchymal stem cells inhibit the skin homing of CD8^+^/CLA^+^ T cells. [64,65]. Further studying and identifying the suitable target molecules, cells and pathways shall help achieve the stability in vitiligo.

## 3. Repigmenting Treatments

A recent global consensus concluded >80% repigmentation to be a successful outcome measure for efficacy of a treatment modality. Moreover, >80% of the repigmentation thus achieved should be maintained for >6 months to tag a modality successful in inducing repigmentation [66]. Repigmentation modalities either replace melanocytes in the vitiliginous skin via surgical procedures [67] (not in the scope of the present review) or enhance the regenerative potential of existing melanocytes. Notably, the stabilization therapies discussed previously have some repigmentation potential by virtue of the arrest of ongoing assault against melanocytes [68]. Also, few patients (1–25%) can have spontaneous repigmentation as well, most commonly seen in children on sun-exposed sites [28]. The sites affected by chronic mechanical friction (fingertips, perioral area) are more prone to melanocyte dislodgement and melanocytorrhagy and show poor response to repigmentation therapies [28].

The repigmentation in vitiligo has been seen to appear in varied morphological patterns, namely, perifollicular, diffuse, marginal and medium spotted [69]. The perifollicular pattern arises chiefly from the proliferation and migration of the melanocytic stem cells located in the bulge area of the outer root sheath of hair follicles to the epidermis. Hair follicles are considered immune privileged sites and therefore, the melanocytic stem cells residing here are preferentially spared from the immunological insult going on at the rest of the sites. The migration of melanocytic stem cells from the hair follicles requires the presence of matrix metalloproteinases (MMP). The expression and activity of metalloproteinases requires the presence of transcription factor Ets-1. A study by Kumar and colleagues revealed that basal levels of Ets-1, MMP-2 and MMP-9 are reduced significantly in vitiligo [70]. Therapeutic implication of MMP induction needs to be studied further. A study evaluated dermapen, fractional carbon dioxide laser and 25% trichloroacetic acid (TCA) in inducing repigmentation in depigmented patches of vitiligo and found that 25% TCA performed the best. The efficacy was suggested to be correlating with an increase in tissue matrix metalloproteinase-9 levels [71].

Understandably, the involvement of these follicular melanocytes/precursors, as evident clinically from the development of leucotrichia, adversely affects the disease prognosis and repigmentation potential. The anatomical sites lacking hair follicles (acral areas, mucosae) are difficult to repigment. Instead of perifollicular, these sites have been shown to gain repigmentation by diffuse, marginal and medium spotted patterns, indicating the potential role of melanocytic stem cells residing in the interfollicular epidermal compartment and dermis [61].

Alpha melanocyte stimulating hormone (α-MSH) has inherent melanocyte stimulating properties. It is synthesized in the keratinocytes after exposure to ultra-violet radiation. The damage created by ultra-violet radiation activates p53, which further mediates transcription of pro-opiomelanocortin (POMC), which is cleaved to form local melanocortin or α-MSH. α-MSH acts on its receptor (melanocortin1 receptor) and activates adenylate cyclase, which causes formation of cyclic adenosine monophosphate (cAMP) from adenosine triphosphate (ATP). The subsequent activation of protein kinase A by cAMP brings about the activation of microphthalmia associated transcription factor (*Mitf)* and c-AMP response element binding protein (*CREB*), both of which have potential pro-differentiation and pro-survival effects on melanocytes. Melanocortin also lowers the oxidative stress inside the melanocytes [48]. The synthetic analogue of α-MSH, afamelanotide, has been found to enhance the repigmentation potential of narrow-band ultra-violet B (NB-UVB). The hyperpigmentation observed in the surrounding non-lesional skin may be worrisome for some patients [4]. Topical placental extract, phenylalanine and beta fibroblast growth factor have been found to be beneficial in inducing repigmentation in conjunction with phototherapy. Beta fibroblast growth factor is released from the keratinocytes on exposure to NB-UVB and promotes melanocyte migration via induction of phosphorylated focal adhesion kinase [72].

Prostaglandins were seen to cause trichomegaly, periorbital hyperpigmentation and darkening of eyelashes when used for the treatment of glaucoma. Since then, these have been utilized for the treatment of vitiligo [73,74,75]. In vitro studies reported a proportional increase in the dendricity of melanocytes and tyrosinase activity when these were incubated with increasing concentrations of fluprostenol. Proliferation of melanocytes was not affected however [73]. A recent study compared a combination of 0.03% bimatoprost and NB-UVB with NB-UVB alone and found the combination to be more beneficial in causing repigmentation. [76] 

Another pathway that has been of recent interest in vitiligo involves the activation of wnt-beta catenin signaling. This signaling is vital for differentiation of melanoblasts into functioning melanocytes and was found to be getting impaired on exposure to oxidative stress [77]. The wnt agonists have been demonstrated to enhance melanocyte differentiation in ex-vivo studies. Moreover, the inhibition of glycogen synthase kinase beta, an important negative regulator of wnt signaling, was found to have an effect analogous to the use of wnt agonists. Also, dermal fibroblasts from hands and feet were found to secrete wnt-inhibitors and that might be responsible for difficulty to achieve satisfactory repigmentation at these sites [4]. One of the plausible mechanisms of action of phototherapy is the induction of wnt signaling [4]. These molecular targets could be utilized in the development of novel therapeutic targets for causing successful repigmentation in vitiligo [78].

## 4. Stabilizing and Repigmenting Treatments

There are few therapies that both stabilize the disease and cause successful repigmentation. The foremost of these include phototherapeutic modalities. Ultra-violet radiation has the maximum potential to cause melanocyte differentiation [4]. The keratinocyte-melanocyte loop works efficiently to protect the human integument from the harmful effects of solar radiation. Exposure of skin to ultra-violet (UV) radiation causes cellular damage in keratinocytes. The damage activates p53 in keratinocytes and it further mediates the transcription and release of beta- fibroblast growth factor (beta-FGF), alpha-melanocyte stimulating hormone (α-MSH), endothelin-1 and adrenocorticotrophic hormone from keratinocytes in a paracrine fashion. These mediators bring about some immediate and late responses in melanocytes.

Immediate responses involve p53-mediated enhanced survival. The late responses include NB-UVB-mediated transcription of microphthalmia-associated transcription factor (MITF), which in turn leads to upregulation of the receptors for endothelin B and c-*KIT* on the melanocyte surface [79]. MITF thus mediates increase in melanogenesis and the transfer of melanin to keratinocytes, resulting in enhanced photoprotection. UV radiation also causes mitosis of melanocytes and results in activation, proliferation and migration of dormant melanocytes residing in the outer root sheath of hair follicles. The effects of UV radiation are also marked on immune effector cells. Overall, this modality results in the halting of depigmentation and initiation of repigmentation in vitiliginous skin and is one of the most successful treatment options for vitiligo, both as monotherapy and combination therapy [80,81,82,83].

Phototherapy includes sunlight, UVA (ultra-violet A), PUVA (psoralen plus UVA), BB-UVB (broadband ultra-violet B), NB-UVB (narrowband UVB) and excimer. NB-UVB is polychromatic with maximum emission between 311-312 nm. NB-UVB has been shown to cause apoptosis of T-cells in active disease. By inducing IL-10, it stimulates the differentiation of Tregs, which also inhibit the autoreactive cytotoxic T-cells [84,85,86,87]. The biostimulation of functional melanocytes in the perilesional skin and immature melanocytes in hair follicles results in repigmentation after treatment with NB-UVB.

The active epidermal melanocytes in vitiligo are selectively targeted by the immune system, whereas the immature/inactive ones residing in the outer root sheath of hair follicles are spared. NB-UVB causes activation, proliferation and migration of melanocytes from perilesional skin and hair follicles. The exposure to NB-UVB increases the expression of endothelin-1 and basic fibroblast growth factor by keratinocytes, which promotes melanocyte growth and proliferation. It also enhances the phosphorylated focal adhesion kinase and matrix metalloproteinase-2 activity in melanocytes that helps in their migration towards depigmented zones [88]. NB-UVB also reduces the oxidative stress in vitiligo. Higher doses of NB-UVB may be required to achieve stabilization than repigmentation [81].

In a prospective study, overall repigmentation with PUVA was 44%, and that with NB-UVB was 52%, when administered over 5.6 and 6.3 months, respectively. Excluding hands and feet, the repigmentation with NB-UVB was 67% and that with PUVA was 54%, and the difference was significant, asserting the beneficial role of NB-UVB as a monotherapy in the treatment of vitiligo [81]. It has also been observed that the response to phototherapy is better in recent onset vitiligo than the long-standing one (>1–2 years of disease). The involvement of melanocytes in hair follicles (characterized by leucotrichia, which is usually a later phenomenon in non-segmental vitiligo than the segmental vitiligo), adversely affects the response to phototherapy. Therefore, early treatment is advocated to delay/avoid the development of leucotrichia [84].

Phototherapy is also an excellent modality to induce stability in active vitiligo. It takes around 3.5 months to induce stability as per vitiligo disease activity (VIDA) scoring (VIDA score 0) [84]. NB-UVB is more efficacious than PUVA in inducing stability and producing repigmentation in active disease. Moreover, a recent meta-analysis states that long-term NB-UVB (12 months) induced maximum (>75%) repigmentation rates. The face and neck were the sites that showed maximum repigmentation, followed by the trunk and limbs. Acral areas did not show >75% repigmentation. Greater than 50% and 75% repigmentation response to NB-UVB at sites other than the face and neck did not change significantly by addition of topical vitamin D3 analogues or topical calcineurin inhibitors [83].

Excimer laser has a monochromatic wavelength of 308 nm. The irradiance of excimer is more than that of NB-UVB. Irradiance is more important than fluence for melanoblast differentiation. This explains the greater efficacy of excimer than NB-UVB for treatment of localized vitiligo. It has been shown to have a greater inhibitory effect on T-cells than NB-UVB and causes maximum T-cell apoptosis and endothelin secretion, of all UVB modalities [6]. It has been observed that though the repigmentation is faster with more frequent treatments, the ultimate degree of repigmentation achieved depends upon the total number of treatments rather than on the frequency. Excimer lamp/monochromatic excimer light has the advantage of having a larger treatment field as compared to excimer lasers.

Apart from their inhibitory action on T-cells, topical calcineurin inhibitors increase the activity of tyrosinase and promote melanogenesis. Tacrolimus stimulates the expression of MITF and melanocyte migration. In fact, the induction of melanocyte migration by tacrolimus is much more significant than that induced by endothelin 1 [79,89]. These have special utility in the treatment of facial lesions, where corticosteroids can induce atrophy of thinner skin. Tacrolimus 0.1% ointment has been seen to have an efficacy equal to the 0.05% clobetasol propionate [28]. The combination of tacrolimus with excimer has been shown to be effective in the treatment of acral vitiligo, which is typically UV-resistant. Its combination with NB-UVB enhances repigmentation rates compared to NB-UVB alone, but a meta-analysis did not support this observation. Though around 14% patients do not respond to these agents, these have overall a good efficacy and tolerability. Moreover, continued application of tacrolimus 0.1% ointment twice a week has been seen to prevent the re-development of vitiligo at previously treated patches, in a pattern akin to that seen with ‘weekend therapy’ of topical calcineurin inhibitors in atopic dermatitis [28]. The results with pimecrolimus are variable. Its effects are said to be more superior on the face than elsewhere [28].

To conclude, vitiligo follows an unpredictable disease course. Quiescent disease can become active, and currently, there are no established biomarkers to predict the course of the disease in an individual. Most of the treatments only provide partial and temporary relief. However, the treatment should always be offered to the patients instead of dismissing it as a simple cosmetic problem. The psychological stress and social ramifications of this disorder can be significant, and timely and adequate treatment is needed. Ongoing basic and translational research shall hopefully reveal promising targets for novel drug development for this disfiguring disease.
